# Analysis on the Epidemiological Characteristics of Breakthrough Varicella Cases and Incremental Effectiveness of 2-Dose Varicella Vaccine in China

**DOI:** 10.3390/vaccines13020160

**Published:** 2025-02-06

**Authors:** Mingzhu Lin, Tian Yang, Pengfei Deng, Laibao Yang, Caoyi Xue

**Affiliations:** Shanghai Pudong New Area Center for Disease Control and Prevention, Shanghai 200136, China; mzlin@pdcdc.sh.cn (M.L.); tyang@pdcdc.sh.cn (T.Y.);

**Keywords:** varicella, 2-dose varicella vaccination strategy, varicella effectiveness, breakthrough varicella

## Abstract

Background: A 2-dose varicella vaccination strategy has been implemented in Shanghai, China since 2018. This study aims to analyze the epidemiological characteristics of breakthrough varicella cases and to evaluate the incremental effectiveness of the 2-dose varicella vaccination among Chinese children. Methods: A retrospective investigation was conducted among native children born between 2015 and 2019 in the Pudong New area of Shanghai, China. From 2016 to 2023, demographic information and data regarding varicella vaccination were collected through the Shanghai Immunization Information System, while information on varicella infections was obtained from the China Information System for Disease Control and Prevention. The incremental vaccine effectiveness (VE) for varicella was defined as (1 − relative risk (RR)) × 100%, where RRs were calculated based on the rate of varicella infections. Results: A total of 519,951 local children were enrolled in the cohort analysis. The overall rate of breakthrough varicella infections was found to be 0.25%, corresponding to 1182 cases. Specifically, the rates of breakthrough varicella infections for individuals who received 1-dose and 2-dose VarV were 0.39% (977 cases) and 0.10% (205 cases), respectively. The average ages of onset for these infections were 2.13 ± 0.99 years and 5.52 ± 1.18 years, respectively. Furthermore, the breakthrough varicella infection rate among individuals born between 2015 and 2019 exhibited a decline, decreasing from 0.52% to 0.26% for those who received one dose of VarV, and from 0.23% to 0.01% for those who received two doses. The VE against all varicella was 85% (95% confidence interval: 84–86) for one dose and 96% (95% confidence interval: 96–97) for two doses, and the incremental VE was 75% (95% confidence interval: 71–79) compared with 1-dose. Conclusions: the 2-dose VarV vaccination strategy provided excellent protection to prevent varicella, and the universal 2-dose regimen of the varicella vaccine should be recommended to prevent varicella disease among children in China.

## 1. Introduction

Varicella, caused by the Varicella-zoster virus (VZV), is an acute, highly infectious disease that spreads primarily through respiratory droplets and direct contact. The typical symptoms include systemic papules and pruritic varicella rashes, which are common in children and can lead to serious complications, such as pneumonia, encephalitis and even death [1]. Contagiousness begins 1–2 days before the onset of the disease and lasts until the rash has reached the dry and crusted stage, with transmission caused by exposure to air droplets from infected individuals [2]. The disease can occur in all seasons of the year, but is more prevalent in winter and spring [3,4], which can easily cause clustered epidemics in schools and kindergartens [5].

Global data from the World Health Organization show that at least 1.4 billion new cases of varicella are reported annually, with 4.2 million cases resulting in serious complications and about 4200 deaths related to varicella [6]. Recent studies have shown that the number of VZV infections worldwide increased by 17.85% in 2019 compared to 1990 [7].The burden of disease caused by varicella is also high. A study in the United States found that varicella-related illness costs 90 million in medical costs per year and 4.39 billion in unemployment costs [8]. A study on the economic evaluation of varicella in Spain [9], analyzed from a social point of view, found that the total cost of varicella treatment was about 1.723 billion dollars over a 50-year period for unvaccinated individuals. In the absence of universal varicella vaccination, 31 European countries carried an annual economic burden of more than 6.6 billion euros [10].

The varicella vaccine (VarV) is the most effective way to prevent and control varicella [11]. The World Health Organization advises routine childhood immunization in countries experiencing a significant public health impact due to the disease [1]. In the United States, hospitalizations and outpatient visits to varicella patients decreased by 88% and 59%, respectively after the varicella vaccine was first included in the National Immunization Program (NIP) as a single-dose regimen between 1994 and 2012 [12]. In Brazil, a single dose of the varicella vaccine was introduced for children at 15 months of age in 2013, and three years later the rate of hospitalizations for varicella among the vaccinated population decreased by 47.6%. The varicella vaccine is 93% effective in preventing severe varicella and the average effectiveness of varicella prevention is 86% [13].

In 1998, China introduced the self-paid varicella vaccine [14], which is considered an effective measure to prevent and control the spread of the disease. Long-term epidemiological surveillance has shown that a certain proportion of breakthrough varicella cases may continue to occur after one dose of VarV vaccination in children, and the incidence rate shows an upward trend as vaccination time increases. For example, a 14-year follow-up study in California, USA, found that the average annual incidence of breakthrough varicella cases was 15.9 ‰ [15,16]. In 2012, a 2-dose VarV immunization strategy was implemented in parts of China, demonstrating good protective effects. However, breakthrough cases of varicella still occurred [15]. The clinical symptoms of varicella breakthrough cases are usually mild [17], but can still be transmitted and cause varicella public health emergencies [18]. Different immunization strategies require different amounts of financial support, and the decision between one or two doses remains a key consideration. To better prevent varicella, Shanghai began to implement emergency vaccination with VarV at schools and kindergartens in 2014 [19]. In 2018, a vaccination strategy involving two free doses of VarV was implemented in Shanghai. There have been no studies on breakthrough cases of varicella in Shanghai so far. This study aims to analyze the epidemiological characteristics of varicella breakthrough cases after one and two doses of VarV in children born in Shanghai between 2015 and 2019. Additionally, it aims to examine the incremental effectiveness of 2-dose varicella vaccination among Chinese children, providing a reference for optimizing the strategy of VarV immunization.

## 2. Methods

### 2.1. Varicella Surveillance

Pudong New Area is located in the eastern part of Shanghai Municipality, China. With approximately 5.81 million residents, Pudong accounts for 20% of Shanghai’s total population. The demographic characteristics in Pudong, such as age distribution, align with those of Shanghai, as indicated in the national census. Furthermore, since the disease surveillance system and immunization program in Pudong are implemented in accordance with the relevant regulations of Shanghai Municipality, the data presented in this study can serve as a reliable representation of the entire city. Since 2006, local health providers and physicians have been required to report varicella cases electronically through the China Information System for Disease Control and Prevention (CISDCP) within 24 h. A voluntary two-dose varicella vaccination schedule has been recommended in Shanghai, China, since November 2017 and was incorporated into the immunization program of Shanghai in August 2018. The vaccination records of children are maintained in the Shanghai Immunization Information System, which includes demographic and vaccination information.

### 2.2. Varicella Vaccination

Shanghai began implementing emergency vaccinations with VarV in schools and kindergartens in 2014. Prior to 2018, one dose of the varicella vaccine was recommended for children aged 12 months in Shanghai. However, starting in August 2018, the two free doses of VarV are respectively administered at 12–28 months of age and again at 4 years of age. Children born on or after 1 August 2014, are eligible for a free second dose of VarV after they reach 4 years of age, while those born before 1 August 2014, may receive a second dose of VarV for a fee.

### 2.3. Data Sources

This study included local children born in the Pudong New Area of Shanghai from 2015 to 2019. Demographic information and varicella vaccination data for these children, covering the period from 2016 to 2023, were collected through the Shanghai Immunization Information System. Additionally, varicella infection information data were obtained from the China Information System for Disease Control and Prevention. It is important to note that during the collection of vaccination data, we included not only children who received vaccinations according to the routine schedule but also those who received their first dose of the varicella vaccine more than 12 months after births with a delay of up to 3 years. This particular group was included because our research aims to comprehensively analyze the association between the varicella vaccination history and the incidence of varicella. The vaccination status of these children provides more detailed information for the study, facilitating an in-depth exploration of the differences in varicella incidence associated with varying vaccination doses. All children included in the study were categorized based on the number of varicella vaccine doses they received. We separately recorded and analyzed the incidence of varicella in children who received only one dose compared to those who received two doses of the varicella vaccine, in order to evaluate the impact of different vaccination doses on the incidence of varicella.

### 2.4. Definitions

Breakthrough varicella is defined as a case that develops more than 42 days after vaccination. The breakthrough infection rate is defined as the proportion of breakthrough infections among children who have received varicella vaccinations.

### 2.5. Data Analysis

The basic characteristics were compared between the breakthrough cases of individuals who received one dose and those who received two doses, using Pearson’s Chi-square test, Fisher’s exact test or the Wilcoxon rank-sum test, as appropriate. The incremental vaccine efficacy (VE) was defined as (1 − relative risk (RR)) × 100%, where RRs were calculated based on the breakthrough infection rates. Recipients of the one-dose vaccine served as the reference group for calculating incremental VE, which was defined as the additional protection conferred by the two-dose vaccination compared to the one-dose vaccination. Data were entered into Microsoft Excel 2010, and statistical analyses were performed using SPSS version 22.0. A *p* value of less than 0.05 was considered statistically significant.

## 3. Results

### 3.1. Population Description

**Figure 1:**
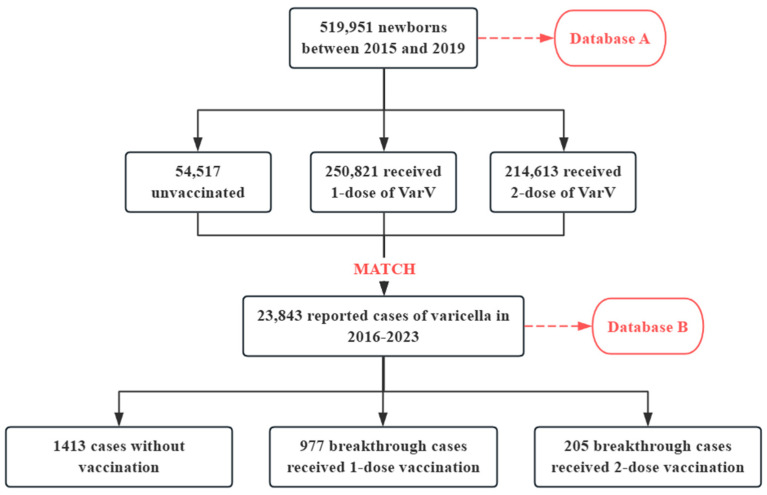
Flowchart for breakthrough varicella cases inclusion.

Data on a total of 519,951 newborns in the Pudong New Area of Shanghai, born between 2015 and 2019, including demographic information and VarV vaccination information, were obtained from the Shanghai Immunization Information System, referred to as “Database A”. Within Database A, 54,517 children did not have varicella vaccination, 250,821 children had received 1-dose of VarV and 214,613 had received 2-dose of VarV. Additionally, 23,843 cases of varicella with onset dates ranging from 2016 to 2023 were obtained through the China Information System for Disease Control and Prevention, referred to as “Database B”. After matching Database A and B based on name, gender and date of birth, a total of 1182 breakthrough varicella cases were obtained, of which 977 cases received one dose of VarV, while 205 cases received two doses of VarV (Figure 1).

### 3.2. Overall Breakthrough of Varicella Infection

**Figure 2:**
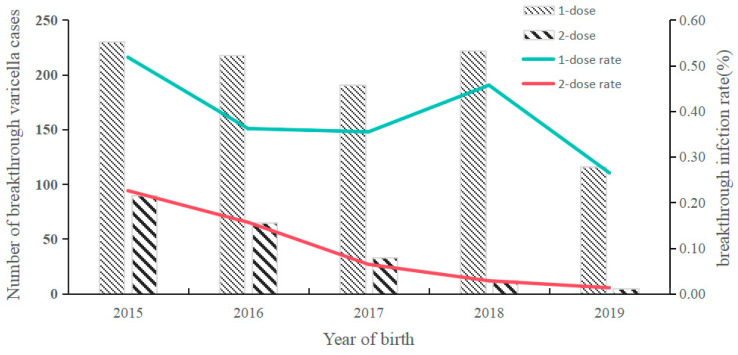
The number of breakthrough varicella cases and breakthrough infection rate among different years of birth.

The overall rate of breakthrough varicella infections among children born between 2015 and 2019 was 0.25%, corresponding to 1182 cases. The rate for children who received 1-dose and 2-dose VarV were 0.39% (977 cases) and 0.10% (205 cases), respectively. Specifically, the breakthrough varicella infection rates for 1-dose recipients by year of birth from 2015 to 2019 were as follows: 0.52%, 0.36%, 0.35%, 0.46% and 0.26%. In contrast, the rates for 2-dose recipients were 0.23%, 0.16%, 0.06%, 0.03% and 0.01% (Figure 2). In each year of birth, the 1-dose vaccinated children had a significantly higher percentage of varicella infection than the 2-dose vaccinated children (*p* < 0.0001). Furthermore, the infection rates for both 1-dose and 2-dose vaccinated children demonstrated a declining trend (Figure 2).

### 3.3. Comparison of Basic Characteristics

The proportion of breakthrough cases following 1-dose from 2016 to 2023 was recorded at 0.82%, 7.68%, 19.24%, 22.11%, 14.43%, 22.00%, 9.52% and 4.20%. In contrast, the proportion of breakthrough cases following two doses between 2019 and 2023 was 4.39%, 8.78%, 28.29%, 22.44% and 36.10%. The average ages of onset for breakthrough cases with 1-dose and 2-dose vaccination were 2.13 ± 0.99 years and 5.52 ± 1.18 years, respectively (*p* < 0.0001). The distribution of breakthrough cases by gender for the 1-dose vaccination was 59.06% male and 40.94% female, while for the 2-dose vaccination, it was 64.44% male and 37.56% female. Statistically significant differences were observed in the year of onset, age and location between breakthrough cases associated with 1-dose vaccination and those associated with 2-dose vaccination (all *p* < 0.05) (Table 1).

**Figure 3:**
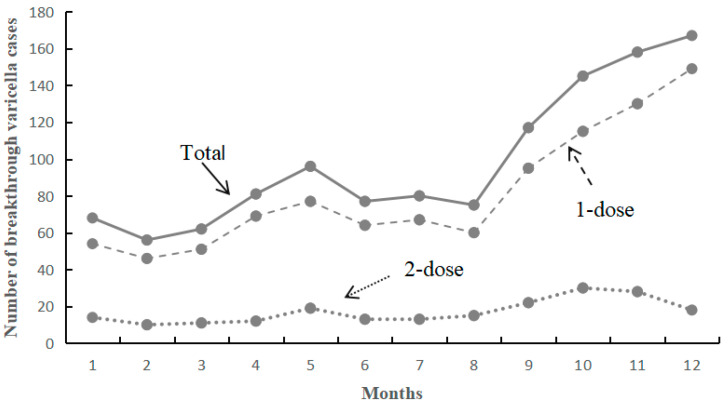
The number of breakthrough varicella cases among the children in different months of onset.

The incidence of breakthrough varicella cases among children born between 2015 and 2019 varied monthly, with reported cases ranging from 56 to 167. Specifically, the number of breakthrough cases among those who received one dose ranged from 46 to 149, while those who received two doses ranged from 10 to 30. The proportion of breakthrough cases for 1-dose and 2-dose recipients from September to December was 50.05% and 47.80%, respectively (Figure 3).

### 3.4. Time Interval Between VarV Vaccination and Onset

**Figure 4:**
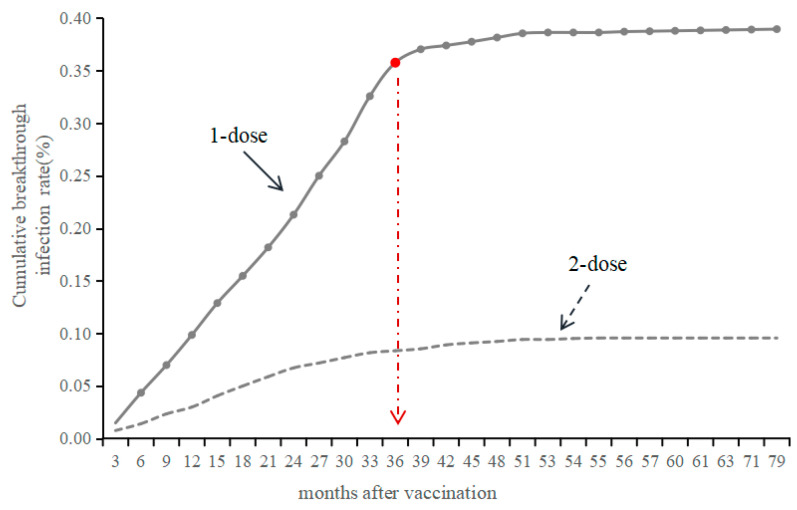
The time interval between VarV vaccination and onset among the children birth of 2015–2019. The dashed red line/arrow represents an inflection point, the 36th month after vaccination. After this point, the trend of the breakthrough infection rate has remained stable.

The longest interval observed between vaccination and the onset of 1182 breakthrough varicella cases was 80.49 months, while the shortest interval was 1.49 months, with a median duration of 22.70 months. For breakthrough varicella cases following 1-dose and 2-dose vaccinations, the intervals between vaccination and onset were recorded as 1.49 to 80.49 months and 1.56 to 56.69 months, respectively. The median intervals were 23.79 months and 18.35 months. The cumulative breakthrough infection rate in 1-dose vaccination increased rapidly to 0.36 within the first 36 months, followed by a gradual increase. In contrast, the 2-dose group exhibited a slow increase to 0.08 within 36 months, after which it stabilized (Figure 4).

### 3.5. Vaccine Effectiveness

The total varicella vaccine effectiveness (VE) of 1-dose VarV was 85% (95% confidence interval: 84–86) and of 2-dose VarV was 96% (95% confidence interval: 96–97). The VE of 1-dose VarV in the five birth cohorts was lower than that of 2-dose VarV, with a minimum value of 73% and a maximum value of 91%. The VE of 2-dose VarV increased year by year, with a minimum of 93% and a maximum of 99%. The incremental varicella vaccine effectiveness (VE) of 2-dose varicella vaccination regimen, in comparison to the 1-dose regimen, exhibited an increase for individuals born between 2015 and 2019, rising from 56% (95% confidence interval: 44.43–65.87) to 95% (95% confidence interval: 88.16–98.02). Furthermore, the overall incremental VE of the 2-dose regimen relative to the 1-dose regimen was determined to be 75% (95% confidence interval: 71.49–78.91). Specifically, the incremental VE for individuals born in 2018 and 2019 was found to be 94% (95% confidence interval: 89.00–96.56) and 95% (95% confidence interval: 88.16–98.02), respectively (Table 2).

## 4. Discussion

The results indicated that the cumulative incidence of breakthrough varicella cases among children born in the Pudong New Area of Shanghai from 2015 to 2019, during the period from 2016 to 2023, was 0.25%. Furthermore, the incidence of breakthrough varicella cases in children who received one or two doses of the varicella vaccine (VarV) increased with the duration of VarV vaccination. The findings demonstrated that the 2-dose VarV was significantly more effective than the 1-dose VarV in preventing varicella infection, with an incremental VE of 75% (confidence interval: 71.49–78.91). Additionally, the incremental VE of 2-dose VarV among children born in 2018 and 2019 was 94% (confidence interval: 89.00–96.56) and 95% (confidence interval: 88.16–98.02), respectively.

The incidence of breakthrough varicella infections among individuals born between 2015 and 2019 has demonstrated a decline, decreasing from 0.52% to 0.26% for those receiving a single dose of the varicella vaccine, and from 0.23% to 0.01% for those receiving two doses. Previous studies [20,21] have indicated that breakthrough varicella cases can occur following both 1-dose and 2-dose VarV, with the incidence of breakthrough varicella cases being lower in individuals who received two doses compared to those who received one dose [22]. This finding aligns with the results of current study, which further suggests that the protective efficacy of 2-dose VarV is superior to that of 1-dose VarV. The protective effect of the varicella vaccine diminishes over time, eventually leading to breakthrough varicella cases. Research conducted in the United States has demonstrated that varicella outbreaks and breakthrough varicella cases still occur following one dose of VarV vaccination, and indicated that even with high coverage of VarV vaccination, the protective effect was approximately 85%, which was insufficient to prevent varicella outbreaks in educational settings such as schools and kindergartens [23,24]. Consequently, it is imperative to revise the vaccination strategy for varicella in China.

The results indicated that the vaccine effectiveness (VE) of 1-dose VarV ranged from 73% (66–77%) to 91% (89–92%), with a mean effectiveness of 84.2%, which is consistent with similar studies conducted in China [4]. This study was performed in a community-based environment characterized by high natural exposure and transmission rates of varicella, where exogenous exposure may contribute to a prolonged enhancement of immunity in vaccinated individuals, potentially leading to an overestimation of the efficacy of one dose of vaccination [25]. In contrast, the VE for 2-dose VarV ranged from 93% (92–95%) to 99% (98–100%) with a mean effectiveness of 96.8%. These results algin with findings from a study conducted in the United States [26], where the one-dose VarV vaccination program was initiated in 1996, followed by the introduction of the two-dose VarV vaccination program in 2006. The first dose was administered at 12–15 months of age, while the second dose was given at 4–6 years of age [27]. The protective effect of the two-dose VarV strategy in the United States was reported to be between 94% and 98% [23]. Additionally, a study by Kauffmann and colleagues further corroborated that the two-dose VarV strategy demonstrated greater effectiveness compared to the one-dose VarV strategy in Germany [28]. An Italian study showed [29] that the most effective strategy was two doses of the varicella vaccine, which caused a 66% reduction in varicella cases and a 30% reduction in varicella-related deaths compared to without VarV immunization strategy, which showed that varicella vaccination played a significant role in reducing varicella incidence and social costs, supporting the Italian policy of varicella vaccine into the NIP.

This study indicated that the majority of breakthrough cases with 1-dose and 2-dose vaccinations occurred in males. It is possible that boys have greater opportunities for exposure to the virus. However, some research has demonstrated that there is no significant gender difference in varicella infection rates [30], while other studies have reported a higher incidence of varicella in females compared to males [7]. These discrepancies may be attributed to varying exposure risks associated with different genders in distinct geographical regions. Additionally, the existing literature suggests that treatment bias and sample collection methods may also contribute to observed gender differences between males [31] and females, as varicella zoster virus is considered a non-gender-specific disease [32].

The incidence of breakthrough varicella cases exhibits distinct seasonal patterns, with elevated rates observed from April to June and September to December [33]. The incidence of breakthrough varicella cases among individuals who received 1-dose and 2-dose VarV was significantly higher during these months, accounting for 50.05% and 47.80%, respectively. The period from April to June and September to December coincide with the academic calendar, during which susceptible populations congregate indoors, thereby increasing the risk of infection. This phenomenon may be attributed to the clustering characteristics of susceptible individuals. Furthermore, related research indicates that varicella is more likely to manifest in winter and spring within warm tropical regions, as the climatic conditions during these seasons facilitate the proliferation and transmission of the varicella virus [34].

Several studies have indicated that the median time interval between varicella vaccination and the occurrence of breakthrough varicella cases is approximately 7 years, with the shortest interval reported being 1.5 years [20]. These intervals are notably longer than the findings of the present study, which may be attributable to factors such as the age at which vaccination was administered and the duration of observation for the study participants. Additionally, another investigation revealed that the time interval between the administration of 1-dose VarV and the onset of varicella was 2.3 years [35], a finding that aligns closely with the results of the current study. In this study, we observed that the cumulative reported incidence of breakthrough varicella cases following one dose of VarV increased rapidly to 0.36 within the first 36 months, subsequently exhibiting a gradual disease. In contrast, the incidence following 2-dose VarV increased slowly to 0.08 within 36 months and then stabilized. Notably, the cumulative breakthrough infection rate in 1-dose VarV was significantly higher than that in 2-dose, indicating that the 2-dose was much more effective in delaying the onset of breakthrough varicella cases compared to the 1-dose VarV. These findings suggest that administering two doses of VarV could potentially reduce the incidence of breakthrough varicella cases, which aligns with previous conclusions indicating that the protective effect of the vaccination in two doses is superior to that of the 1-dose vaccination [21,36,37]. Furthermore, the incremental VE of 56% and 57% for 2-dose VarV in individuals born in 2015 and 2016 was considerably lower than the incremental VE of 94% and 95% observed in those born in 2018 and 2019. This discrepancy may be partially attributed to the waning of immunity or an increased risk of exposure among older children [38] (those born in 2015–2016 were 7–8 years old at the end of 2023), which also suggests a trend of decreasing VE over time.

This study acknowledges several limitations. The incidence of varicella breakthrough cases across various birth cohorts was only monitored until the conclusion of 2023. Consequently, the cumulative rates at different intervals post-vaccination were not comprehensively documented. Additionally, some breakthrough varicella cases exhibited varying clinical symptoms. Mild or atypical infections may not prompt individuals to seek medical attention, which could result in an underestimation of the incidence of breakthrough varicella cases.

## 5. Conclusions

In conclusion, the incidence of breakthrough varicella infections among individuals who received 2-dose vaccination was significantly lower than that observed in those who received one dose of the varicella vaccine. Additionally, the effectiveness of the 2-dose vaccine exceeded 90% in comparison to the 1-dose vaccine, indicating that the 2-dose vaccination schedule provides substantially greater protection than the 1-dose vaccination schedule. Therefore, it is recommended that the expanded 2-dose regimen of the varicella vaccine be advocated for the prevention of varicella infections among children in China.

## Figures and Tables

**Table 1:** Comparison of basic characteristics between breakthrough cases with 1-dose and 2-dose varicella vaccination among children born between 2015 and 2019.

Basic Characteristics	Breakthrough Cases with 1-Dose (N = 977)	Breakthrough Cases with 2-Dose (N = 205)	*p* Value
Year of onset			*p* < 0.0001
2016	8 (0.82)	0	
2017	75 (7.68)	0	
2018	188 (19.24)	0	
2019	216 (22.11)	9 (4.39)	
2020	141 (14.43)	18 (8.78)	
2021	215 (22.00)	58 (28.29)	
2022	93 (9.52)	46 (22.44)	
2023	41 (4.20)	74 (36.10)	
Age	2.13 ± 0.99	5.52 ± 1.18	*p* < 0.0001
Gender			0.207
Male	577 (59.06)	128 (64.44)	
Female	400 (40.94)	77 (37.56)	
Location			0.035
Local	773 (79.12)	174 (84.88)	
Inward	204 (20.88)	31 (15.12)	

**Table 2:** The overall and incremental vaccine effectiveness of 2-dose varicella vaccination among the children born between 2015 and 2019.

Year of Birth	Vaccination Status	N	Case	RR (95% CI)	VE % (95% CI)
2015	Unvaccinated	8511	288	Reference	
	1-dose	44,381	230	0.15 (0.13–0.18)	85 (82–87)
	2-dose	39,877	90	0.07 (0.05–0.08)	93 (92–95)
	Incremental *			0.44 (0.34–0.56)	56 (44–66)
2016	Unvaccinated	11,092	423	Reference	
	1-dose	60,252	218	0.09 (0.08–0.11)	91 (89–92)
	2-dose	41,587	65	0.04 (0.03–0.05)	96 (95–97)
	Incremental *			0.43 (0.32–0.57)	57 (43–67)
2017	Unvaccinated	10,410	301	Reference	
	1-dose	53,828	191	0.12 (0.10–0.15)	88 (85–90)
	2-dose	51,484	33	0.02 (0.02–0.03)	98 (97–98)
	Incremental *			0.18 (0.12–0.26)	82 (74–88)
2018	Unvaccinated	12,557	205	Reference	
	1-dose	48,578	222	0.27 (0.23–0.34)	73 (66–77)
	2-dose	42,665	12	0.02 (0.01–0.03)	98 (97–99)
	Incremental *			0.06 (0.03–0.11)	94 (89–97)
2019	Unvaccinated	11,947	196	Reference	
	1-dose	43,782	116	0.16 (0.13–0.20)	84 (80–87)
	2-dose	39,000	5	0.01 (0.003–0.02)	99 (98–100)
	Incremental *			0.05 (0.02–0.12)	95 (88–98)
Total	Unvaccinated	54,517	1413	Reference	
	1-dose	250,821	977	0.15 (0.14–0.16)	85 (84–86)
	2-dose	214,613	205	0.04 (0.03–0.04)	96 (96–97)
	Incremental *			0.25 (0.21–0.28)	75 (71–79)

* 1-dose recipients as reference.

## Data Availability

Restrictions apply to the availability of these data, which were collected from the Shanghai Pudong and National Information System. Data are available from the authors with permission from the Shanghai Pudong New Area Center for Disease Control and Prevention.
